# A Diagnostic Challenge: Severe Thrombocytopenia During Streptococcal Cellulitis Revealing Previously Unrecognized Immune Thrombocytopenia

**DOI:** 10.7759/cureus.113949

**Published:** 2026-08-04

**Authors:** Alireza Izadian Bidgoli, Alberto Gomez Usatorres, Hellen Ailette Gomez Usatorres, Alberto Gomez Veliz

**Affiliations:** 1 Internal Medicine, American University of the Caribbean School of Medicine, Cupecoy, SXM; 2 Internal Medicine, Jackson Memorial Hospital, Miami, USA; 3 Internal Medicine, Hispano-American University, San José, CRI

**Keywords:** cellulitis, corticosteroid therapy, immune thrombocytopenia, platelet transfusion refractoriness, streptococcus pyogenes

## Abstract

Severe thrombocytopenia is frequently encountered in patients with acute bacterial infections and is often attributed to infection-associated platelet consumption, potentially delaying recognition of alternative etiologies. We report the case of a 72-year-old man with chronic unexplained thrombocytopenia who presented with fever, generalized weakness, and progressive bilateral upper-extremity cellulitis, predominating in the left forearm where a chronic nonhealing fishhook injury had become secondarily infected, while the contralateral forearm demonstrated diffuse erythema, warmth, and swelling without an associated wound. Initial evaluation demonstrated leukocytosis, markedly elevated inflammatory markers, and profound thrombocytopenia with a platelet count of 12 ×10^3^/µL. Imaging confirmed left forearm cellulitis without abscess formation or necrotizing soft tissue infection, and wound cultures grew *Streptococcus pyogenes*. The left forearm infection improved with targeted intravenous antimicrobial therapy, while the contralateral inflammatory changes gradually resolved; however, severe thrombocytopenia persisted despite multiple platelet transfusions. Further hematologic evaluation demonstrated a persistently elevated immature platelet fraction, giant platelets on peripheral smear, negative peripheral blood flow cytometry, and no laboratory evidence of thrombotic microangiopathy or disseminated intravascular coagulation, favoring peripheral immune-mediated platelet destruction over bone marrow failure or consumptive thrombocytopenia. High-dose corticosteroid therapy produced a rapid and sustained platelet recovery, confirming immune thrombocytopenia. This case highlights the importance of reconsidering the differential diagnosis when severe thrombocytopenia persists despite resolution of concurrent soft tissue infections. Failure to respond to platelet transfusions, together with evidence of preserved platelet production and corticosteroid responsiveness, should prompt early consideration of immune thrombocytopenia and facilitate timely initiation of appropriate immunosuppressive therapy while avoiding unnecessary diagnostic procedures and ineffective transfusion strategies.

## Introduction

Immune thrombocytopenia (ITP) is an acquired autoimmune disorder characterized by isolated thrombocytopenia resulting from immune-mediated platelet destruction and impaired platelet production. It remains a diagnosis of exclusion because no single laboratory test definitively confirms the disease, requiring careful integration of clinical history, physical examination, peripheral blood smear, and targeted laboratory investigations to exclude secondary causes of thrombocytopenia [1-3].

The differential diagnosis of severe thrombocytopenia is broad and includes infection-associated thrombocytopenia, disseminated intravascular coagulation, thrombotic microangiopathies, drug-induced thrombocytopenia, hypersplenism, nutritional deficiencies, and bone marrow disorders [1-4]. Distinguishing ITP from these conditions is particularly challenging in hospitalized patients with acute bacterial infections, where thrombocytopenia is frequently attributed to the infectious process or consumptive mechanisms [1,3].

Current international guidelines recommend corticosteroids as first-line therapy for adults with newly diagnosed ITP who require treatment [1,2]. However, recognizing ITP before initiating immunosuppressive therapy remains challenging when competing etiologies for thrombocytopenia coexist. Evidence of preserved platelet production, including an elevated immature platelet fraction (IPF) and the presence of giant platelets on peripheral blood smear, together with poor response to platelet transfusion, may provide important clues supporting immune-mediated peripheral platelet destruction [1,2,4].

We report the case of a 72-year-old man admitted with *Streptococcus pyogenes* cellulitis and profound thrombocytopenia that was initially attributed to the acute infection. Persistent thrombocytopenia despite appropriate antimicrobial therapy and repeated platelet transfusions prompted a comprehensive hematologic evaluation. The subsequent rapid and sustained response to corticosteroid therapy ultimately established the diagnosis of previously unrecognized ITP, highlighting the importance of avoiding diagnostic anchoring and of reassessing the differential diagnosis when platelet kinetics are inconsistent with the presumed underlying illness.

## Case presentation

Clinical presentation

A 72-year-old man with a history of hypertension, prediabetes, and chronic unexplained thrombocytopenia presented to the emergency department with a two-day history of generalized weakness, fever, chills, and progressive swelling of both upper extremities, accompanied by worsening pain, erythema, and purulent drainage from the left forearm. Approximately two years earlier, he had sustained a fishhook injury to the left forearm while fishing, resulting in a chronic nonhealing wound with intermittent drainage. Several days before the presentation, he struck the affected arm against a box, after which the wound became increasingly erythematous, swollen, and painful. In addition to the chronic left forearm lesion, physical examination demonstrated diffuse erythema, warmth, and edema of the right forearm, which was more swollen than the left but notably nontender and without drainage or an identifiable portal of entry. He also reported progressive difficulty ambulating because of generalized weakness but denied chest pain, dyspnea, spontaneous bruising, epistaxis, gingival bleeding, hematuria, melena, or other bleeding manifestations.

The patient reported that thrombocytopenia had first been recognized several months before admission. According to prior medical records, his platelet count had declined from approximately 80-100 ×10^3^/µL five months earlier to approximately 37 ×10^3^/µL three months later. He had previously undergone hematologic evaluation at an outside institution, where bone marrow biopsy had been recommended but was never performed. His home medications included valsartan and metformin. Tirzepatide had recently been discontinued after thrombocytopenia was identified because of concern for a possible drug-related etiology.

Initial assessment and physical examination

**Figure 1:**
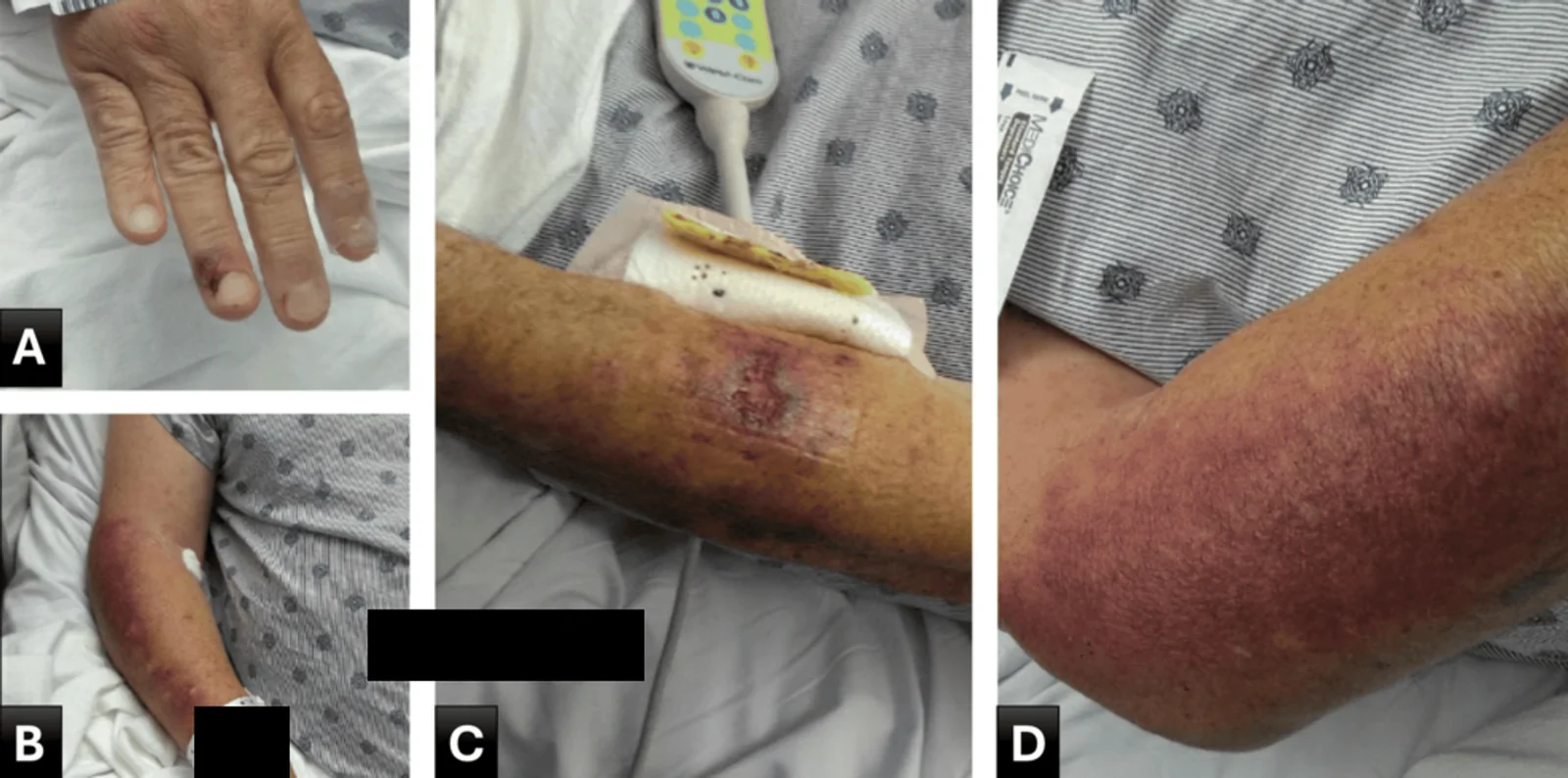
Clinical appearance of the upper extremities at hospital admission. (A) Hemorrhagic lesion involving the right ring finger. (B) Diffuse erythema and swelling extending along the right forearm. (C) Chronic ulcerative lesion over the dorsal mid-forearm at the site of a previous fishhook injury with surrounding cellulitis on the left upper extremity. (D) Close-up view illustrating marked erythema, edema, and induration surrounding the chronic wound of the right upper extremity. These findings were present on admission before initiation of antimicrobial therapy.

On presentation, the patient was febrile (38.4°C) but remained hemodynamically stable. Physical examination revealed a 2 × 2 cm chronic ulcerated wound over the dorsal aspect of the left forearm with surrounding erythema, warmth, edema, and purulent drainage, consistent with cellulitis. No palpable fluctuance, drainable fluid collection, crepitus, or clinical evidence of compartment syndrome was identified. Examination of the contralateral upper extremity demonstrated diffuse erythema, warmth, and edema involving the right forearm, which appeared more swollen than the left but was notably nontender and without an associated wound or drainage. Distal motor strength, sensation, radial pulses, and capillary refill were preserved bilaterally. No focal neurologic deficits were appreciated, and the remainder of the physical examination was unremarkable. Representative clinical photographs obtained at admission demonstrate the asymmetric bilateral upper-extremity inflammatory changes, with the chronic infected left forearm wound and diffuse right forearm edema and erythema (Figure 1).

Orthopedic hand surgery evaluated the patient shortly after admission. Cross-sectional imaging and clinical examination demonstrated no drainable abscess, necrotizing soft tissue infection, or acute orthopedic pathology requiring operative intervention. The patient was therefore managed nonoperatively with intravenous antimicrobial therapy and close clinical monitoring.

Laboratory and diagnostic testing

Initial laboratory evaluation demonstrated leukocytosis (15.4 ×10^3^/µL) and profound isolated thrombocytopenia with a platelet count of 12 ×10^3^/µL. Hemoglobin was 13.7 g/dL with a mean corpuscular volume of 80.7 fL. The IPF was markedly elevated at 28%, indicating preserved marrow platelet production. Inflammatory markers were significantly elevated, with a C-reactive protein of 19.9 mg/dL and an erythrocyte sedimentation rate of 98 mm/hr, while serum lactate was mildly elevated at 3.1 mmol/L. Coagulation studies demonstrated an international normalized ratio (INR) of 1.41, activated partial thromboplastin time of 34 seconds, and elevated fibrinogen of 610 mg/dL, findings not consistent with disseminated intravascular coagulation. Lactate dehydrogenase (176 U/L), haptoglobin (287 mg/dL), bilirubin, and liver function tests were within normal limits, arguing against thrombotic microangiopathy or significant hemolysis. Serologic testing for human immunodeficiency virus (HIV) and hepatitis C virus was negative (Table 1).

**Table 1:** Summary of laboratory findings during hospitalization. Summary of the patient's laboratory findings at admission, throughout hospitalization, and at discharge. The patient presented with profound isolated thrombocytopenia associated with a markedly elevated IPF, indicating preserved bone marrow platelet production despite severe thrombocytopenia. Inflammatory markers were elevated in the setting of acute *Streptococcus pyogenes* cellulitis, whereas coagulation parameters, hemolysis markers, liver function tests, and infectious serologies were largely unremarkable, helping exclude disseminated intravascular coagulation, thrombotic microangiopathy, and secondary infectious causes of thrombocytopenia. Despite appropriate antimicrobial therapy and repeated platelet transfusions, thrombocytopenia persisted until initiation of corticosteroid therapy, after which platelet counts began to recover, supporting the diagnosis of immune thrombocytopenia. ALT: alanine aminotransferase; AST: aspartate aminotransferase; aPTT: activated partial thromboplastin time; CRP: C-reactive protein; ESR: erythrocyte sedimentation rate; HIV: human immunodeficiency virus; HCV: hepatitis C virus; IPF: immature platelet fraction; INR: international normalized ratio; LDH: lactate dehydrogenase

Laboratory Test	Admission	Range During Hospitalization	Discharge	Reference Range (Unit)
White blood cell count	15.4	14.2-22.9	18.3	4.0-11.0 ×10^3^/µL
Hemoglobin	13.7	12.4-13.7	12.7	13.5-17.5 g/dL
Platelet count	12	11-159	159	150-400 ×10^3^/µL
IPF	28	14-42	11.2	1.0-7.0%
ESR	98	45-98	-	<20 mm/hr
CRP	19.9	13.0-32.5	-	<0.5 mg/dL
Lactate	3.1	1.7-3.1	-	0.5-2.0 mmol/L
INR	1.41	1.29-1.41	-	0.8-1.2
aPTT	34	29-34	-	25-35 sec
Fibrinogen	610	610-862	-	200-400 mg/dL
LDH	176	176-258	-	120-250 U/L
Haptoglobin	287	258-287	-	30-200 mg/dL
Total bilirubin	1.4	0.3-1.0	0.3	0.2-1.2 mg/dL
AST	25	25-47	47	10-40 U/L
ALT	25	18-38	38	7-56 U/L
HIV 1/2 Ag/Ab	Negative	Negative	-	Negative
HCV antibody	Negative	Negative	-	Negative

**Figure 2:**
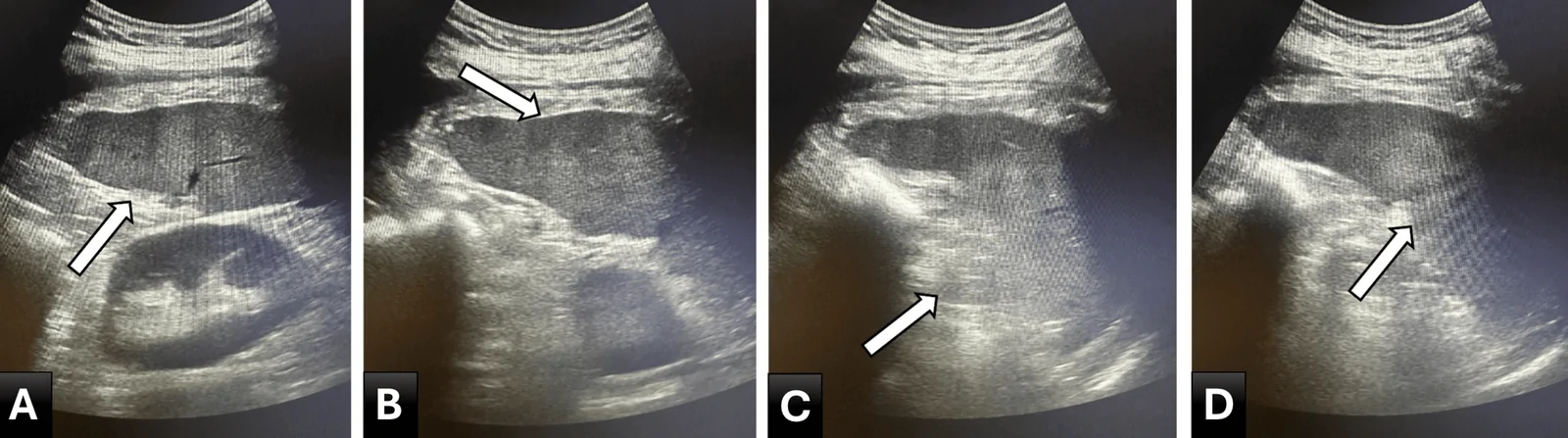
Abdominal ultrasonography demonstrating mild splenomegaly. Longitudinal grayscale ultrasonographic images of the spleen obtained during the diagnostic evaluation. Images (A-D) demonstrate diffuse splenic enlargement (white arrows), with a maximal splenic length of approximately 14.9 cm, consistent with mild splenomegaly. No focal splenic lesion, infarction, perisplenic fluid collection, or other sonographic abnormalities were identified. The finding prompted further hematologic evaluation in the setting of persistent severe thrombocytopenia and supported investigation for an underlying immune-mediated process.

Bilateral upper-extremity venous duplex ultrasonography demonstrated no evidence of deep venous thrombosis or other clinically significant vascular abnormality. Abdominal ultrasonography performed as part of the hematologic evaluation demonstrated mild splenomegaly measuring 14.9 cm (Figure 2) without focal splenic lesions or other significant sonographic abnormalities.

Despite progressive improvement of the cellulitis following antimicrobial therapy, the patient's severe thrombocytopenia persisted. Platelet transfusion produced only a minimal transient increase in platelet count (12 ×10^3^/µL to 13 ×10^3^/µL). Repeat hematologic evaluation demonstrated a further rise in the IPF from 28% to 42%, reinforcing ongoing peripheral platelet destruction. Peripheral blood flow cytometry showed no increase in myeloid blasts or immunophenotypically abnormal B- or T-cell populations.

Multidisciplinary board discussion

The patient's care involved close collaboration among Internal Medicine, Hematology/Oncology, Infectious Diseases, and Orthopedic Hand Surgery.

Initially, the profound thrombocytopenia was believed to be multifactorial in the setting of acute bacterial cellulitis. The differential diagnosis included infection-associated consumptive thrombocytopenia, drug-induced thrombocytopenia, nutritional deficiency, myelodysplastic syndrome, other primary bone marrow disorders, and ITP. Given the patient's several-month history of progressive thrombocytopenia predating the current infection, an underlying hematologic disorder remained an important consideration.

Orthopedic Hand Surgery excluded a surgical process requiring debridement, while the Infectious Diseases service directed antimicrobial therapy after superficial wound cultures grew *S. pyogenes*. As the bilateral upper-extremity cellulitis steadily improved with appropriate antimicrobial therapy, the patient's severe thrombocytopenia persisted despite platelet transfusions, making isolated infection-associated thrombocytopenia increasingly unlikely.

Hematology integrated the persistently elevated IPF, giant platelets on peripheral smear, negative peripheral blood flow cytometry, absence of laboratory evidence supporting thrombotic microangiopathy or disseminated intravascular coagulation, poor response to platelet transfusions, and chronic history of thrombocytopenia. Importantly, the patient had isolated thrombocytopenia without additional cytopenias, and peripheral blood flow cytometry demonstrated no immunophenotypically abnormal myeloid, B-cell, or T-cell populations, reducing the likelihood of leukemia, lymphoproliferative disorders, or other primary bone marrow pathology. Furthermore, the markedly elevated IPF suggested preserved bone marrow thrombopoiesis rather than impaired platelet production. Because the overall clinical and laboratory findings favored peripheral immune-mediated platelet destruction, immediate bone marrow biopsy was not pursued, and empiric corticosteroid therapy was initiated. The subsequent rapid and sustained platelet recovery strongly supported a clinical diagnosis of ITP.

Diagnosis and management

The patient was initially treated with empiric intravenous vancomycin and cefepime for presumed bacterial cellulitis. Following wound culture results demonstrating *S. pyogenes*, antimicrobial therapy was narrowed to intravenous cefazolin, resulting in progressive resolution of the soft tissue infection.

Although infection improved clinically, platelet counts remained critically low despite transfusion support. The combination of chronic thrombocytopenia, persistently elevated IPF (28% increasing to 42%), giant platelets on peripheral smear, normal flow cytometry, absence of evidence for disseminated intravascular coagulation or thrombotic microangiopathy, and refractoriness to platelet transfusions strongly favored ITP.

Based on the evolving clinical picture, high-dose prednisone (80 mg daily; approximately 1 mg/kg/day) was initiated as empiric therapy for presumed ITP while continuing close hematologic monitoring.

Clinical outcome

**Figure 3:**
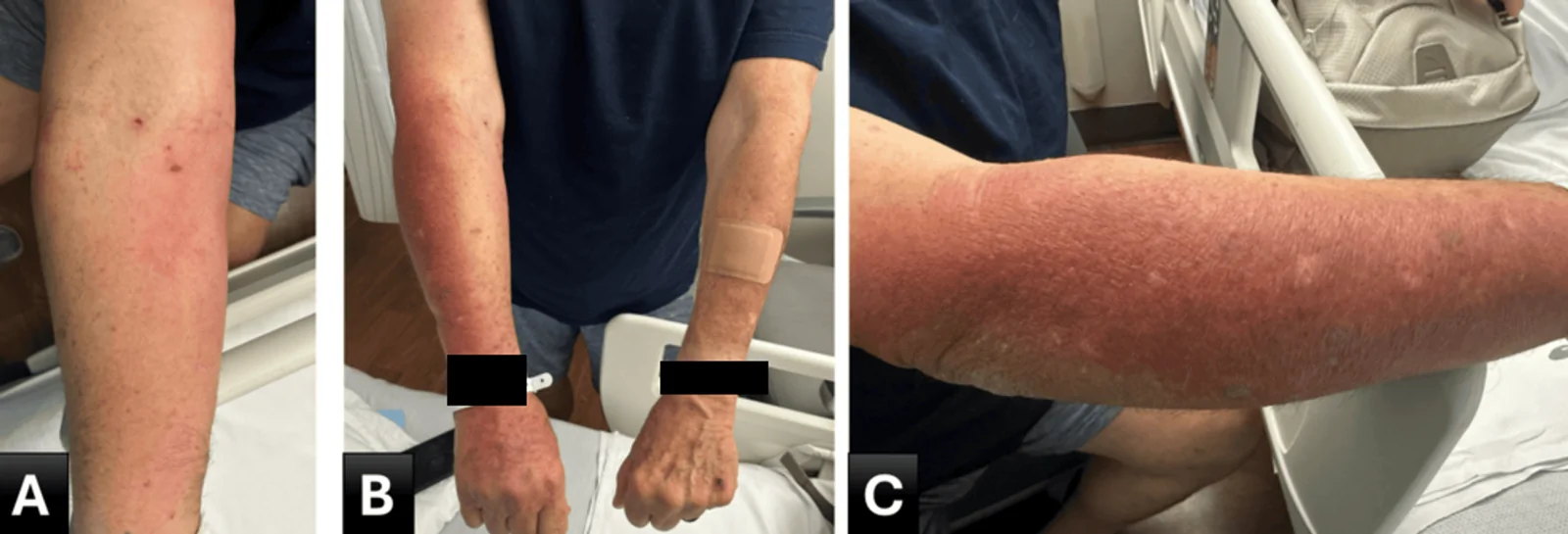
Clinical improvement of the upper extremities following treatment. (A) Resolution of erythema surrounding the left dorsal forearm with healing of the chronic wound. (B) Bilateral upper extremities demonstrating substantial reduction in swelling and inflammation of the affected right forearm. (C) Close-up view showing significant improvement in erythema and induration with no evidence of progressive soft tissue infection of the right upper extremity.

Following initiation of high-dose corticosteroid therapy, the patient's platelet count demonstrated a rapid and sustained recovery, increasing from 12 ×10^3^/µL on admission to 159 ×10^3^/µL at discharge, strongly supporting the diagnosis of ITP. Concurrently, the bilateral upper-extremity soft tissue infection responded favorably to targeted antimicrobial therapy. The left forearm cellulitis showed progressive resolution of erythema, edema, and induration, with interval healing of the chronic dorsal forearm wound and no evidence of recurrent purulence or progression of infection. The contralateral right forearm erythema and edema also improved substantially, with resolution of induration and no development of neurovascular compromise (Figure 3). No additional surgical intervention was required, and no clinically significant bleeding complications occurred during hospitalization.

The patient remained hemodynamically stable throughout the remainder of his hospital course and was discharged home on an oral prednisone taper and oral cephalexin to complete treatment for *S. pyogenes* cellulitis. Close outpatient follow-up with hematology was arranged for continued monitoring of platelet recovery and long-term management of newly diagnosed ITP.

## Discussion

Background

ITP, formerly known as idiopathic thrombocytopenic purpura or Werlhof disease, is an acquired autoimmune disorder characterized by isolated thrombocytopenia (platelet count <100 × 10^9^/L) resulting from immune-mediated platelet destruction and impaired platelet production within the bone marrow [5,6]. The disease was first described in 1735 by the German physician Paul Gottlieb Werlhof, who documented a patient with spontaneous mucocutaneous bleeding, establishing the first clinical description of what is now recognized as ITP [7].

ITP is an uncommon hematologic disorder with an estimated annual incidence of 1.6-3.9 cases per 100,000 persons, and its incidence increases with age. The disease demonstrates a bimodal age distribution, affecting younger women more frequently during early adulthood, whereas older adults are affected equally or with a slight male predominance [6,8]. Approximately 80% of adult cases are classified as primary ITP, while the remaining cases are secondary to conditions such as autoimmune diseases, lymphoproliferative disorders, viral infections (particularly HIV and hepatitis C), medications, and, less commonly, vaccinations [6].

Clinical manifestations range from asymptomatic thrombocytopenia to petechiae, purpura, ecchymoses, mucosal bleeding, and, rarely, life-threatening hemorrhage [8]. Because no definitive diagnostic test exists, ITP remains a diagnosis of exclusion requiring careful evaluation to eliminate alternative causes of thrombocytopenia, including thrombotic microangiopathy, disseminated intravascular coagulation, bone marrow disorders, infections, and drug-induced thrombocytopenia [6].

Pathology and pathophysiology

ITP is an autoimmune disorder characterized by a loss of immune tolerance to platelet surface glycoproteins (GPs), most commonly GPIIb/IIIa and GPIb/IX [1,5]. Autoantibodies produced by autoreactive B lymphocytes bind circulating platelets, promoting Fc receptor-mediated phagocytosis by splenic macrophages and accelerated platelet destruction within the reticuloendothelial system [1,9]. In addition to enhanced peripheral destruction, platelet production is impaired because autoantibodies and cytotoxic T lymphocytes directly damage megakaryocytes and inhibit thrombopoiesis in the bone marrow [1,10].

The adaptive immune response plays a central role in disease pathogenesis. Dysregulation of T-helper cells, reduced regulatory T-cell activity, increased cytotoxic CD8⁺ T-cell responses, and abnormal cytokine signaling perpetuate autoantibody production and immune-mediated platelet clearance [5,10]. Although thrombopoietin concentrations increase in response to thrombocytopenia, the rise is insufficient to compensate for ongoing platelet destruction and impaired megakaryocyte function [1]. Consequently, patients typically present with isolated thrombocytopenia, while coagulation studies and other hematologic parameters remain normal unless an alternative or concomitant disorder is present [1,9]. Recognition of these immunologic mechanisms has led to targeted therapies, including thrombopoietin receptor agonists, anti-CD20 monoclonal antibodies, and spleen tyrosine kinase inhibitors, in addition to traditional immunosuppressive treatment [1].

Comparative analysis with the current literature

Clinical Presentation

Primary ITP most commonly presents as isolated thrombocytopenia with mucocutaneous bleeding, petechiae, ecchymoses, or is discovered incidentally during routine laboratory evaluation [1,11]. Although bleeding severity generally correlates with platelet count, many adults remain asymptomatic despite profound thrombocytopenia, particularly when platelet counts decline gradually [1,11]. In contrast, our patient sought medical attention because of acute *S. pyogenes* cellulitis rather than bleeding complications, despite a platelet count of only 12 ×10^3^/µL. Importantly, review of his prior laboratory studies revealed progressive thrombocytopenia several months before hospitalization, suggesting that the immune-mediated process preceded the infectious episode. The concurrent bacterial infection initially favored a diagnosis of infection-associated thrombocytopenia, creating a diagnostic challenge not commonly emphasized in contemporary ITP literature [1,12].

Diagnostic Workup

Current international guidelines emphasize that ITP remains a diagnosis of exclusion because no single laboratory test confirms the disease [1]. Instead, diagnosis relies on excluding secondary causes of thrombocytopenia through careful clinical assessment, peripheral blood smear review, and targeted laboratory evaluation. Our patient's diagnostic workup closely followed this approach. Coagulation studies, fibrinogen, lactate dehydrogenase, bilirubin, and haptoglobin were inconsistent with disseminated intravascular coagulation or thrombotic microangiopathy, while HIV and hepatitis C serologies were negative. Peripheral blood flow cytometry excluded an underlying hematologic malignancy, and peripheral smear demonstrated giant platelets without schistocytes, findings characteristic of peripheral platelet destruction rather than impaired marrow production [1,3].

An additional distinguishing feature was the persistently elevated IPF, which increased from 28% to 42% during hospitalization. Recent studies have demonstrated that elevated IPF has important diagnostic value in differentiating ITP from hypoproliferative thrombocytopenias because it reflects preserved megakaryocyte activity despite accelerated peripheral platelet destruction [4]. Although IPF should not be used in isolation, its interpretation alongside platelet transfusion refractoriness and peripheral smear findings substantially strengthened the clinical suspicion for ITP in our patient.

Management

Current American Society of Hematology and international consensus guidelines recommend corticosteroids as first-line therapy for adults with newly diagnosed ITP requiring treatment, with intravenous immunoglobulin reserved for patients with active bleeding or those requiring rapid platelet recovery [1,13]. Platelet transfusions are generally reserved for life-threatening hemorrhage or invasive procedures because transfused platelets undergo the same immune-mediated destruction as endogenous platelets [1].

Our patient's management illustrates this principle. Initially, repeated platelet transfusions produced only minimal and transient improvement despite appropriate treatment of the cellulitis. As infection improved but thrombocytopenia persisted, the combination of chronic platelet decline, elevated IPF, giant platelets, and poor transfusion response shifted the diagnostic consideration toward ITP. Initiation of high-dose prednisone resulted in a rapid and sustained increase in platelet count, serving both as effective treatment and as strong clinical confirmation of immune-mediated platelet destruction.

Outcome and Follow-Up

Although our patient achieved a complete platelet response following corticosteroid therapy, long-term remission after first-line treatment cannot be assumed. Corticosteroids remain the standard initial therapy for newly diagnosed ITP and achieve an initial platelet response in most patients; however, relapse during corticosteroid tapering or after treatment discontinuation is common, necessitating continued hematologic surveillance [2,4,11,13]. Current international guidelines therefore emphasize close outpatient follow-up after the initial response to detect recurrent thrombocytopenia before the development of clinically significant bleeding [2,13].

Serial complete blood count (CBC) monitoring should be individualized according to the patient's platelet trend, bleeding risk, and clinical stability, with more frequent assessments during corticosteroid tapering and gradually increasing intervals once a sustained platelet response has been established [2,12]. Patients should also receive education regarding the signs and symptoms of recurrent thrombocytopenia and major bleeding, including petechiae, spontaneous bruising, mucosal bleeding, hematuria, melena, or persistent severe headache suggestive of intracranial hemorrhage, and should be instructed to seek immediate medical attention if these occur [2,11]. In the event of relapse, repeat evaluation should exclude secondary causes of thrombocytopenia and guide escalation to second-line therapies, including thrombopoietin receptor agonists, rituximab, fostamatinib, or splenectomy, in accordance with current evidence-based recommendations [2,4,11,12].

Literature Review Summary

Recent literature has increasingly emphasized that the diagnosis of ITP requires continuous reassessment rather than reliance on a single laboratory finding [1,3]. Although infection-associated thrombocytopenia is common among hospitalized patients, persistence of severe thrombocytopenia despite adequate treatment of the underlying infection should prompt reconsideration of alternative diagnoses [1,12]. Our case reinforces several diagnostic features consistently associated with ITP, including elevated IPF, giant platelets on peripheral smear, and poor response to platelet transfusion [1,4]. Unlike many published reports describing patients who initially present with bleeding manifestations, our patient's hospitalization for acute cellulitis obscured recognition of chronic ITP. This case therefore highlights the importance of avoiding diagnostic anchoring and demonstrates how integration of clinical history, platelet kinetics, peripheral smear findings, and therapeutic response can facilitate timely diagnosis of ITP even in the presence of competing explanations for thrombocytopenia. Table 2 summarizes the comparative analysis of this case with the current literature.

**Table 2:** Comparison of the present case with selected contemporary studies on ITP. ITP: immune thrombocytopenia; IVIG: intravenous immunoglobulin; IPF: immature platelet fraction; DIC: disseminated intravascular coagulation; TMA: thrombotic microangiopathy

Article Details	Patient(s)	Trigger/Clinical Presentation	Major Diagnostic Findings	Treatment	Outcome and Comparison With the Present Case
Cooper et al., 2019 [11]	Adults with primary ITP	Variable presentation ranging from incidental thrombocytopenia to severe mucocutaneous or life-threatening bleeding	Diagnosis based on isolated thrombocytopenia after exclusion of secondary causes	Corticosteroids ± IVIG; rituximab, thrombopoietin receptor agonists, or splenectomy for refractory disease	Mirrors the diagnostic approach used in our patient, in whom alternative causes were systematically excluded before confirming ITP.
Anat, 2023 [1]	Adults with thrombocytopenia	Thrombocytopenia caused by either peripheral platelet destruction or impaired marrow production	Elevated IPF favored peripheral platelet destruction	Diagnostic evaluation only	Similar to our patient, whose IPF increased from 28% to 42%, supporting preserved marrow production and immune-mediated platelet destruction.
Matzdorff et al., 2023 [12]	Adults with infection-associated ITP	Severe thrombocytopenia following acute infection	Extensive evaluation excluded other causes of thrombocytopenia	IVIG and corticosteroids	Platelet recovery occurred after immunosuppressive therapy rather than platelet transfusion, similar to our patient's minimal transfusion response and rapid steroid-induced recovery.
González-López et al., 2023 [3]	Elderly male with infection-triggered ITP	Profound thrombocytopenia after infection	Diagnosis established after exclusion of DIC and other secondary etiologies	IVIG followed by corticosteroids	Demonstrated infection as an immune trigger and excellent response to corticosteroids, consistent with the clinical course observed in our patient.
Liu et al., 2023 [4]	Adults with newly diagnosed ITP	Severe thrombocytopenia requiring treatment	Standard diagnostic evaluation confirmed primary ITP	First-line glucocorticoids	Reported favorable platelet recovery following corticosteroid therapy, comparable to our patient's platelet increase from 12 ×10^3^/µL to 159 ×10^3^/µL.
Neunert and Cooper, 2018 [13]	Adults with secondary ITP following infection	Infection-associated ITP with severe thrombocytopenia	Diagnosis required exclusion of thrombotic microangiopathy, DIC, marrow disorders, and drug-induced thrombocytopenia	Corticosteroids with or without IVIG	Most patients responded to immunosuppressive therapy rather than platelet transfusion, paralleling the present case.
Present case	72-year-old man	*Streptococcus pyogenes* cellulitis with severe isolated thrombocytopenia	Elevated IPF (28-42%), giant platelets, negative flow cytometry, no DIC/TMA, mild splenomegaly	Antibiotics plus high-dose prednisone	Platelet recovery to 159 ×10^3^/µL with marked clinical improvement, confirming previously unrecognized ITP.

What we learned from this case

This case illustrates the diagnostic challenge of distinguishing ITP from secondary thrombocytopenia in patients presenting with acute bacterial infection. Although severe thrombocytopenia is frequently attributed to infection-related platelet consumption, this patient's thrombocytopenia preceded the infectious episode, persisted despite clinical resolution of cellulitis, and demonstrated minimal response to platelet transfusion. These features prompted reconsideration of the initial diagnosis and highlighted the importance of reassessing the differential diagnosis as new clinical information emerges.

Several findings proved particularly informative. A persistently elevated IPF, giant platelets on peripheral smear, normal peripheral blood flow cytometry, absence of laboratory evidence supporting thrombotic microangiopathy or disseminated intravascular coagulation, and refractoriness to platelet transfusion collectively favored peripheral immune-mediated platelet destruction over impaired marrow production or consumptive coagulopathy. The subsequent rapid platelet recovery following initiation of high-dose corticosteroid therapy further supported the diagnosis of ITP.

This case emphasizes that severe thrombocytopenia in the setting of infection should not automatically be attributed to the infectious process alone, particularly when thrombocytopenia predates the infection or fails to improve as the infection resolves. Careful integration of the patient's clinical history, platelet kinetics, peripheral smear findings, and targeted laboratory evaluation can prevent diagnostic anchoring and facilitate timely recognition of ITP, allowing appropriate immunosuppressive therapy while avoiding unnecessary invasive investigations or ineffective repeated platelet transfusions.

## Conclusions

This case highlights the diagnostic complexity of severe thrombocytopenia occurring in the setting of an acute bacterial infection. Although infection-associated thrombocytopenia is common, persistent and transfusion-refractory thrombocytopenia despite appropriate antimicrobial therapy should prompt reconsideration of the initial diagnosis. In our patient, the combination of chronic thrombocytopenia predating the infectious episode, elevated IPF, giant platelets on peripheral smear, absence of laboratory evidence supporting disseminated intravascular coagulation or thrombotic microangiopathy, and minimal response to platelet transfusion suggested an immune-mediated process. The subsequent rapid and sustained platelet recovery following corticosteroid therapy ultimately confirmed the diagnosis of ITP. This case underscores the importance of avoiding diagnostic anchoring, particularly when thrombocytopenia behaves inconsistently with the presumed underlying illness. Careful reassessment of the clinical course, integration of platelet kinetics with targeted laboratory findings, and recognition of corticosteroid responsiveness can facilitate earlier diagnosis of ITP, prevent unnecessary diagnostic procedures and ineffective transfusion strategies, and enable timely initiation of appropriate therapy.
